# Multicomponent Analysis of Sleep Using Electrocortical, Respiratory, Autonomic and Hemodynamic Signals Reveals Distinct Features of Stable and Unstable NREM and REM Sleep

**DOI:** 10.3389/fphys.2020.592978

**Published:** 2020-12-03

**Authors:** Christopher Wood, Matt Travis Bianchi, Chang-Ho Yun, Chol Shin, Robert Joseph Thomas

**Affiliations:** ^1^Division of Pulmonary, Critical Care and Sleep Medicine, Department of Medicine, Beth Israel Deaconess Medical Center, Boston, MA, United States; ^2^Division of Sleep Medicine, Department of Neurology, Massachusetts General Hospital, Boston, MA, United States; ^3^Department of Neurology, Bundang Clinical Neuroscience Center, Seoul National University Bundang Hospital, Seongnam, South Korea; ^4^Division of Pulmonary, Sleep and Critical Care Medicine, Department of Internal Medicine, Korea University Ansan Hospital, Ansan, South Korea

**Keywords:** bimodal, hemodynamic, coupling, cardiopulmonary, sleep

## Abstract

A new concept of non-rapid eye movement (NREM) and rapid eye movement (REM) sleep is proposed, that of multi-component integrative states that define stable and unstable sleep, respectively, NREM_S_, NREM_US_ REM_S_, and REM_US_. Three complementary data sets are used: obstructive sleep apnea (20), healthy subjects (11), and high loop gain sleep apnea (50). We use polysomnography (PSG) with beat-to-beat blood pressure monitoring, and electrocardiogram (ECG)-derived cardiopulmonary coupling (CPC) analysis to demonstrate a bimodal, rather than graded, characteristic of NREM sleep. Stable NREM (NREM_S_) is characterized by high probability of occurrence of the <1 Hz slow oscillation, high delta power, stable breathing, blood pressure dipping, strong sinus arrhythmia and vagal dominance, and high frequency CPC. Conversely, unstable NREM (NREM_US_) has the opposite features: a fragmented and discontinuous <1 Hz slow oscillation, non-dipping of blood pressure, unstable respiration, cyclic variation in heart rate, and low frequency CPC. The dimension of NREM stability raises the possibility of a comprehensive *integrated multicomponent network model of NREM sleep* which captures sleep onset (e.g., ventrolateral preoptic area-based sleep switch) processes, synaptic homeostatic delta power kinetics, and the interaction of global and local sleep processes as reflected in the spatiotemporal evolution of cortical “UP” and “DOWN” states, while incorporating the complex dynamics of autonomic-respiratory-hemodynamic systems during sleep. Bimodality of REM sleep is harder to discern in health. However, individuals with combined obstructive and central sleep apnea allows ready recognition of REM_S_ and REM_US_ (stable and unstable REM sleep, respectively), especially when there is a discordance of respiratory patterns in relation to conventional stage of sleep.

## Introduction

Although sleep is an integrated brain-body state involving multiple coupled physiological systems, it is still categorized into traditional electroencephalographic (EEG)-based subtypes of rapid eye movement (REM) and non-rapid eye movement (NREM). In humans, the latter state is graded, from stage N1 to N3, based on EEG sleep spindles, K-complexes and delta waves of ≥75 μV in amplitude and 0.5–2.0 Hz in frequency. In human scoring, attempts have been made to differentiate “deep” from “light” NREM sleep, most typically by combining N1 and N2 into the latter, but uncertainty persists. One challenge with the current definitions is that stage N3 requires above-threshold slow wave activity for only 20% of a 30-s epoch, leading to great physiological heterogeneity within epochs scored as stage N3. The cyclic alternating pattern (CAP) is one proposed heuristic for characterizing EEG NREM bistability (Parrino et al., 2014) as an alternative to the traditional grading of NREM stages. Other approaches have attempted to parse NREM sleep into finer delineations with 9 or more sub-stages of NREM sleep (Ogilvie, 2001), and a continuous analysis of sleep power bands [Odds Ratio Product] (Younes et al., 2015).

Infraslow activity patterns are key components of NREM sleep, having a major role in clustering of other oscillatory phenomena such as sleep spindles and slow waves (Lecci et al., 2017). The CAP is based on visually recognized repeated sequences of transient EEG events (CAP A) vs. tonic background EEG patterns (CAP B) on the infraslow time scale that show abrupt variations in their spectral composition (Terzano and Parrino, 2000). CAPs appear preferentially at moments of N2 onset or termination, whereas the 0.02-Hz oscillation runs throughout NREM periods (Fernandez and Luthi, 2020). The relation to sleep spindle dynamics is not fully established, but spindles occur preferentially during the B- rather than the A-phase of CAP (Fernandez and Luthi, 2020). The CAP periods are considered unstable periods of NREMS, during which activity in the periphery is increased and the tendency for sleep interruption is enhanced. Many autonomic parameters, such as heart and breathing rates, are modulated in phase with CAP dynamics. The tendency for arousals, leg movements, and bruxism is also enhanced during CAP periods.

Autonomic physiology presents an alternative window into sleep; e.g., hemodynamics, heart rate variability (HRV), and respiration are markedly sleep stage dependent, with vagal dominance, stable breathing, and blood pressure reductions (“dipping”) during slow wave sleep/N3 (Javaheri and Redline, 2012). However, scored N3 is not a reliable marker of these dynamics, which occur in large segments of scored N2 as well. Coupled with the fact that N3 makes up only a small fraction of NREM sleep, and markedly decreases with age even in healthy adults, this suggests an alternative, multi-physiology approach to sleep stability might be more informative than a legacy frontal EEG-based definition. Further, standard reporting of EEG-based stages as percentage of sleep time is an insensitive metric of sleep fragmentation (Bianchi and Thomas, 2013).

A fundamental electrophysiological signature of NREM sleep is the <1 Hz cortical slow oscillation (SO) and the associated defining “UP” and “DOWN” neuronal states, which is seen in virtually every cortical cell recorded intracellularly (Vyazovskiy and Harris, 2013). The slow oscillation can be recorded at subcortical and brainstem levels, and likely influences neural activity (Eschenko et al., 2012). Generally, the SO is enriched during stage N3, but is also present during stage N2. How does the SO relate to downstream autonomic effects of sleep state?

**FIGURE 1:**
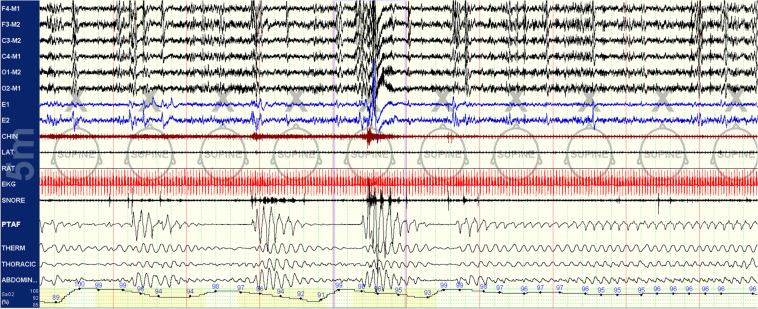
Spontaneous state/respiration switching in NREM sleep. Unstable NREM sleep on the left, spontaneous switch to stable NREM sleep in the middle of this 5-min epoch. EEG montage is standard. LAT, left anterior tibial electromyogram; RAT, right anterior tibial electromyogram; EKG, electrocardiogram; SNORE, snoring microphone; PTAF, pneumotachogram air flow (nasal pressure); THERM, Thermistor airflow; THORACIC, chest effort; and ABDOMIN, abdominal effort.

A method to categorize sleep independent of the EEG, based on cardiopulmonary coupling (CPC) analysis, combines HRV, and the ECG-derived respiration (EDR; Thomas et al., 2005). One regime of NREM sleep is characterized by high frequency cardiopulmonary coupling (HFC), breath-to-breath temporal stability of respiration, relatively high arousal thresholds, strong respiratory sinus arrhythmia, and a paucity of phasic EEG activity; this state we term stable NREM sleep (NREM_S_). High frequency coupling correlates with relative delta power across the entire night, not just N3 rich periods meeting consensus scoring criteria (Thomas et al., 2014). A contrasting regime demonstrates low frequency cardiopulmonary coupling (LFC), low frequency oscillations of tidal volume, cyclic variation in heart rate, abundant phasic EEG activity; we term this integrated state unstable NREM sleep (NREM_US_), because it is associated with known features of sleep fragmentation such as arousals and sleep apnea. These HFC/LFC states are mutually exclusive, switch spontaneously within an individual NREM period, and occur throughout the night (Thomas et al., 2005). These state switches are clearly seen during transitions between stable and unstable NREM breathing in sleep apnea patients (Figure 1). Stable and unstable breathing periods have long been recognized in sleep apnea but have been ascribed to respiratory reflex than top-down brain mechanisms (Jordan et al., 2009; de Melo et al., 2017). An unknown component is the relationship of the NREM slow oscillation to CPC-determined sleep states and that of sleep hemodynamics (blood pressure) to NREM_S_ and NREM_US_. Watson et al. described “packets” of NREM sleep alternating with “microarousals,” interspersed within NREM epochs, characterized by increased firing rates of slow-firing neurons in mice (Watson et al., 2016). It is possible that these “packets” and “microarousals” are the murine counterpart of HFC/LFC switching seen in humans.

Several methods which characterize sleep in alternate ways analyze a single physiological stream (EEG-CAP/non-CAP; EEG-ORP; blood pressure dipping periods; stable breathing periods in sleep apnea). However, there is ample data derived from network physiology research that sleep is a complex vertically (across subsystems) networked state with horizontal (over time of night) dynamics (Bartsch et al., 2015; Liu et al., 2015; Lin et al., 2016, 2020). Across physiological states of “deep” and “light” sleep, the network undergoes topological transitions associated with fast reorganization of multiple diverse physiological interactions on time scales of a few minutes (Bashan et al., 2012). Analysis of Time Delay Stability (TDS), a computational approach to identify and quantify networks of physiologic interactions from long-term continuous, multi-channel physiological recordings, differentiates “deep” and “light” NREM sleep, with far higher connectivity during “light” sleep (Bartsch et al., 2015). Similarly, there are multiple forms of cardiorespiratory coupling (Bartsch et al., 2012), but both autonomic activity and respiration are profoundly influenced by sleep state and thus coupling is markedly different in deeper vs. lighter sleep. Thus, network physiology principles make it entirely plausible that sleep can be bimodal.

In this study, we tested the hypothesis that: (1) slow oscillations would have distinct and different kinetics during NREM_S_ and NREM_US_; (2) blood pressure dipping would occur during NREM_S_, regardless of conventional sleep stage; and (3) REM sleep would demonstrate stable and unstable patterns, specifically that unstable REM sleep would show concomitant features of NREM physiology. Phasic and tonic REM sleep are readily recognized and differentiated by visual examination, but a substantial part of the scoring manual tries to force a 30-s epoch at the border-zone of REM sleep into discrete bins based on elaborate rules for REM/NREM/wake transitions. However, by combining these patterns with respiratory signals, greater clarity of state may be possible.

## Materials and Methods

Three sets of data were used to establish the relationships of NREM_S_ and NREM_US_ with the SO and sleep hemodynamics. Analysis was performed on different data sets for the following reasons: (1) the differentiation of NREM_S_ and NREM_US_ is very clear in patients with sleep apnea – and provides the best resource to establish SO dynamics in relation to electrocardiogram (ECG)-spectrogram defined NREM sleep states; (2) The relationship of HFC and blood pressure profiles is best demonstrated in healthy subjects, so that confounds from autonomic dysfunction seen commonly in sleep apnea patients are avoided; and (3) A third data set focused exclusively on the transition to REM sleep in patients with sleep apnea. IRB approval for retrospective analysis of clinical PSG data was obtained for performance of the studies and data analysis.

### Datasets Overview and Motivation

Three datasets were used to develop the concepts elaborated in this paper, summarized in Table 1. The driving principles of selecting these datasets were as follows: (1) stable and unstable states of NREM sleep are most readily quantified in sleep apnea patients, where the switch from stable to unstable state is so abrupt and with such clear boundaries that it enables capturing segments of relatively pure state. (2) Healthy subjects are optimal for demonstrating hemodynamic associations of sleep state, as states of disrupted sleep may have abnormal autonomic regulation (as is common for example in sleep apnea). (3) The boundary between stable and unstable REM sleep is best seen in those with high loop gain obstructive sleep apnea, with admixed periodic breathing and obstructive pathology (Gilmartin et al., 2005). In these patients with NREM-dominant obstructive sleep apnea, the “central” influence is seen in NREM sleep, and any obstructive component in REM sleep, which eliminates the central component through complex physiological mechanisms. A typical example of “REM_US_” is when obstructive respiratory pathology in NREM sleep stabilizes a few minutes prior the start of REM sleep – the EEG shows NREM sleep, but the respiration shows REM qualities.

**TABLE 1:** Subject characteristics, sleep stage and sleep state across datasets.

**Measure**	**Data-1 (apnea) mean ± SD**	**Data-2 mean ± SD**	**Data-3 (high loop gain apnea) mean ± SD**
Sample size	20	11	50
Age	36.1 ± 6.3	27 ± 2.3 years	54.3 ± 8 years
BMI Kg/M^2^	36.2 ± 3.3	24.8 ± 1.2	32.3 ± 6.7
Gender	13 males	6 males	37 males
Total sleep time (TST, minutes)	380.6 ± 66.4	374.4 ± 61.7	366 ± 31.4
Sleep efficiency	79 ± 12.5	78.7 ± 12.1	85.8 ± 15.2
Wake after sleep onset (minutes)	83.9 ± 51.4	76.2 ± 44.6	52.3 ± 23.9
N1% TST	18.7 ± 14.9	13.8 ± 7.1	15.3 ± 8.3
N1 duration (minutes)	71.2 ± 14.1	48.7 ± 21.8	56.7 ± 18.3
N2% TST	50.1 ± 13.7	46.5 ± 6.5	48.3 ± 7.1
N2 duration (minutes)	228.7 ± 32.8	177 ± 52.3	178.6 ± 41.8
N3% TST	13.1 ± 7.1	22.5 ± 6.8	16.7 ± 9.2
N3 duration (minutes)	49.9 ± 18.2	82.7 ± 23.9	61.1 ± 18.1
REM% TST	17.8 ± 7	17.2 ± 4.8	15.9 ± 11.2
REM duration (minutes)	67.8 ± 11.9	65.8 ± 25.1	58.2 ± 13.7
Arousal index	29.6 ± 22.5	19.2 ± 7.7	38.6 ± 12.8
Apnea-hypopnea index	46.3 ± 7.8	0.7 ± 1	41.7 ± 18.2
HFC% sleep period	32 ± 22.5	49.6 ± 17	–
HFC duration (minutes)	148.632.7	228.581.7	–
LFC% sleep period	43.3 ± 20.9	35.7 ± 14.6	–
LFC duration (minutes)	201 ± 37.2	165.1 ± 73.7	–
VLFC% sleep period	18.9 ± 11.7	14.1 ± 5	–
VLFC duration (minutes)	87.8 ± 17.3	65.3 ± 24.3	–

*TST, total sleep time; N1, N2, N3, standard non-rapid eye movement sleep stages; REM, rapid eye movement; HFC, high frequency coupling; LFC, low frequency coupling; and VLFC, very low frequency coupling.*

### High Frequency Coupling and the Slow Oscillation of NREM Sleep (Data-1)

As the cardiopulmonary states of sleep stability and instability are especially well defined in those with sleep apnea, we analyzed 20 patients from the clinical sleep laboratory with sleep apnea (13 male, age 36.1 ± 6.3 years, body mass index 36.2 ± 3.3 Kg/M^2^, apnea hypopnea index 46.3 ± 7.8/h of sleep). We also selected all night diagnostic PSG from three subjects (2 male, age: 24 ± 3 years, body mass index 24.6 ± 3.2 Kg/M^2^) who presented with fatigue but had normal PSG. Sleep stage, arousal and respiratory event scoring used AASM guidelines^1^ including respiratory event scoring that included respiratory effort related arousals. First, the raw data was reviewed, and tags placed manually to mark periods of at least 10-min duration with the following criteria: (1) The stage was NREM. (2) The slow oscillation was clearly visible after digitally filtering out frequencies greater than 1 Hz. (3) The segments were artifact- and wake-free.

### Slow Oscillation Phenotyping

**FIGURE 2:**
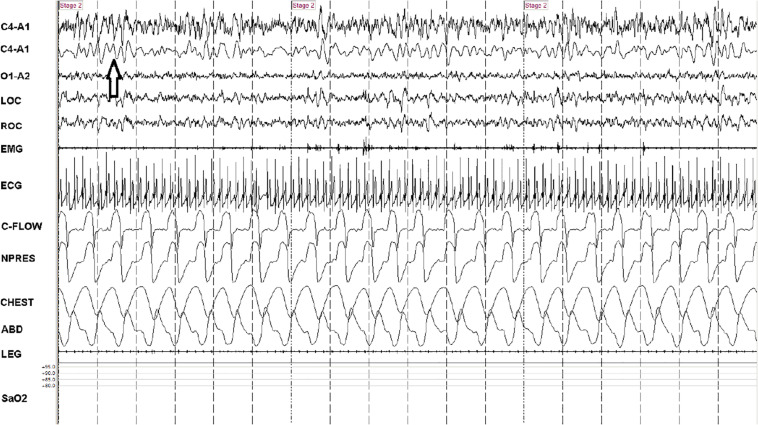
Consolidated/continuous slow oscillation/down states. A 90-s snapshot showing sleep state features of effective NREM, stage N2. Note occurrence of several cycles of the slow oscillation/cortical down states (arrow) within the 5-s vertical time stamps. The second trace (from above) is a duplicate C4-A1 with filters adjusted to remove frequencies >1 Hz. Note strong respiratory modulation of ECG amplitude, which is a key input signal (the ECG-derived respiration) for cardiopulmonary coupling analysis. At this compression, heart rate variability is not readily identified, but the spectrogram is high frequency coupling. Note stable respiration. C-FLOW: flow from positive airway pressure device. NPRESS: nasal pressure. C4, O1: central and occipital standard EEG sites. A: auricle/mastoid reference. EMG: chin electromyogram. LOC/ROC: left and right outer canthus. LEG: combined right and left tibialis anterior electromyogram. SaO_2_: oxygen saturation via finger probe. ECG: electrocardiogram.

**FIGURE 3:**
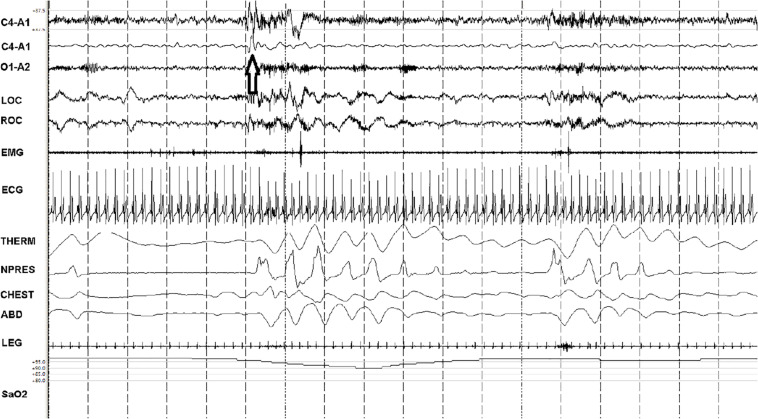
Fragmented slow oscillation and sparse down states. A 90-s snapshot showing sleep state features of ineffective NREM (NREM_IE_), stage N2. Note paucity of the <1 Hz slow oscillation/down states (the arrow identifies an isolated down state, a K-complex) within the 5-s vertical time stamps. The second trace (from above) is a duplicate C4-A1 with filters adjusted to remove frequencies <1 Hz. This is a sample from a patient with untreated sleep apnea, showing the key characteristics of NREM_IE_: a fragmented and sparse slow oscillation, cortical arousals, cyclic variation in heart rate and low frequency modulation of HRV and ECG-amplitude that drives detection of this state as “low frequency coupling.” THERM: thermistor airflow. NPRESS: nasal pressure. C4, O1: central and occipital standard EEG sites. A: auricle/mastoid reference. EMG: chin electromyogram. LOC/ROC: left and right outer canthus. LEG: combined right and left tibialis anterior electromyogram. SaO_2_: oxygen saturation via finger probe. ECG: electrocardiogram.

The C4-A1 channel was selected, and a high frequency filter of 1 Hz used to allow visualization of the <1 Hz slow oscillation during NREM sleep, scored as N1, N2, or N3 in 30 s epochs per standard clinical criteria. Periods of EEG, independent of other physiological signals, were visually characterized in 30-s epochs based on the state determined by the temporal evolution of the slow oscillation, to be either continuous or fragmented throughout the tagged periods. In the continuous mode of the slow oscillation, two EEG cycles with the minimum requirement of 50 μV and 0.1–0.9 Hz were present in every 5 s of the recording. In the fragmented mode, there were at least 5 s without slow waves. Examples are provided in Figures 2, 3. A given 30 s epoch was scored as continuous or fragmented slow oscillation if >50% of a 30-s epoch met the scoring threshold.

### Sleep Spectrograms

The ECG was exported in ASCII or EDF (European Data Format) formats and used to generate sleep spectrograms. Analysis was performed in RemLogic^TM^ (1.1, Embla Systems, now Natus). Briefly, the method is based on simultaneous analysis of HRV and EDR (Thomas et al., 2005). The latter is computed by measuring the variations in QRS complex amplitude due to shifts in the cardiac electrical axis relative to the electrodes during respiration and changes in thoracic impedance as the lungs fill and empty. The HRV and EDR measures are determined in a fully automated way from a single, continuous channel of ECG. A time series of normal-to-normal sinus (NN) intervals and the time series of the EDR associated with these NN intervals are then extracted from the RR interval time series. Fourier-based techniques are employed to analyze the NN interval series and its associated EDR signal. Outliers due to false or missed R-wave detections are removed using a sliding window average filter with a window of 41 data points and rejection of central points lying outside 20% of the window average. The resulting NN interval series and its associated EDR are then resampled at 2 Hz. using cubic spline interpolation. The cross-spectral power and coherence of these two signals are calculated over a 1024 samples (8.5 min) window. Specifically, each 1024 samples window is divided into 3 overlapping 512 samples sub-windows, and Fast Fourier Transform is used to estimate the cross-power and coherence within the 1024 window. The 1024 samples window is then advanced by 256 samples (2.1 min) and the calculation repeated until the entire NN interval/EDR series is analyzed. For each 1024 window the product of the coherence and cross-spectral power is used to calculate the ratio of coherent cross power in the low frequency (0.01–0.1 Hz) band to that in the high frequency (0.1–0.4 Hz) band. A preponderance of power in the low frequency band tends to be associated with periodic sleep behaviors regardless of etiology, while excess power in the high frequency band is associated with physiologic respiratory sinus arrhythmia and stable/deep NREM sleep. Low frequency coupling has been previously shown to be associated with CAP and high frequency coupling with non-CAP (Thomas et al., 2005).

### Beat-to-Beat Blood Pressure and Sleep Stage/State (Data 2)

Polysomnography (PSG) was performed in 11 subjects, 9 were healthy volunteers. Scoring used the AASM guidelines, as above. The PSG system used SOMNOmedics (SOMNOmedics GmbH, Germany) includes Pulse Transit Time derived beat-to-beat blood pressure (Gesche et al., 2012). This was supplemented by intra-arterial blood pressure (2 subjects with hypercapnic sleep apnea monitored in the intensive care unit for accurate titration of positive airway pressure therapy) and Finometer-based beat-to-beat blood pressure in the 9 healthy subjects.

To align results, beat-to-beat blood pressure was averaged every 32 s, so there were 4 readings of beat-to-beat blood pressure for each 128 s moving window of CPC output of HFC vs. non-HFC state. This approach also allowed us to closely track 30 s sleep state epochs, with a small amount of rounding. Thus, each sleep stage epoch had one final reading of blood pressure (systolic or diastolic) and each CPC window had 4 blood pressure readings, which were then averaged.

### Sleep Apnea and REM Border-Zones (Data – 3)

Fifty diagnostic or split-night PSGs of sleep apnea were reviewed to identify coexisting features of REM and NREM sleep but using respiratory patterns (obstructive apnea/hypopnea vs. periodic breathing) as a key guide (Figure 5). Thus, periodic breathing in REM sleep, NREM EEG during REM-type sleep apnea and stabilization of NREM periodic breathing at the edges of REM sleep, before conventional REM sleep, were examples of mixed states identified. Oximetry patterns (band-like desaturation for periodic breathing, V-shaped desaturation for REM-dominant obstructive disease) were also used to identify respiratory sleep stage (Figure 6). These mixed states are called “unstable REM sleep.” The minimum window size used to define this state was 60 s.

### Statistical Methods

Statistical analysis using STATA12 generated summary measures (means/standard deviations), *t*-tests, and repeated measures Analysis of Variance, to assess blood pressure in relation to conventional sleep stage and ECG-spectrogram state.

## Results

### Sleep Spectrogram and NREM Sleep < 1 Hz Slow Oscillation in Data-1

From a total of 20 subjects with sleep apnea and 3 subjects with chronic fatigue, a total of 1256.5 min of sleep was analyzed. There were 35 periods of sleep selected for analysis and scoring, the mean duration was 35.9 ± 21.1 min. The 7 periods visually scored as “fragmented” were 44.6 ± 21.7 min in duration, not significantly different in duration from the 28 periods scored as “continuous,” 33.7 ± 12.3, *t*-test *p* = 0.2. On conventional staging, 9 periods were stage N3 and 26 were stage N2; the N3 periods were significantly longer, 49.2 ± 12.5 vs. 31.3 ± 8.3 min, *t*-test *p* = 0.03.

### Slow Oscillation, Conventional Stage, and High Frequency Coupling

In Data-1, high frequency coupling state dominated sleep periods where the <1 Hz slow oscillation was scored as “continuous.” The continuous mode showed increased high frequency coupling (83.1 ± 8.1 vs. 18.6 ± 6.7% for low frequency coupling, *p* < 0.001). There was no significant difference in HFC% when considering epochs scored by traditional N2 vs. N3 (70.1 ± 39.3% vs. 70.7 ± 40.8%, *p*: 0.97). There were no significant association between <1 Hz slow oscillation modes with conventional stage N2/N3 [Pearson Chi Square: 0.60, *p*: 0.50]. In Data-2, the slow oscillation continuous mode dominated periods of high frequency coupling contrasted with low frequency coupling (93.3 ± 3.4 vs. 12.7 ± 5.6%, *p* < 0.001).

### Sleep Stage, Sleep Spectrogram in Data-2 (Tables 1, 2)

Sleep efficiency was lower and wake after sleep onset higher than that typical of research studies, likely reflecting the recording environment and mild discomfort with the Finometer cuff and intermittent calibration inflations of a blood pressure cuff which is part of the recording system. Adequate numbers of stages and states were obtained (Table 2). There was no significant sleep apnea, including in the two subjects studied during positive airway pressure titration in the intensive care unit.

**TABLE 2:** (Data -2): Epochs of artifact free conventional stage/interpolated CPC.

**Subject**	**N1**	**N2**	**N3**	**REM**	**HFC**	**Non-HFC**
1	48	323	0	54	164	259
2	68	387	51	71	123	454
3	57	290	32	22	328	73
4	19	173	81	118	222	169
5	28	195	170	74	199	286
6	23	406	168	221	154	664
7	35	74	90	0	32	167
8	20	215	139	90	108	356
9	7	394	220	108	322	407
10	40	313	195	78	68	558
11	41	408	125	161	155	508
Total	386	3178	1271	997	1875	3901

*N1, N2, N3, non-rapid eye movement stages; REM: rapid eye movement; HFC: high frequency coupling; and CPC: cardiopulmonary coupling.*

### EEG Delta Power, Sleep Stages, High Frequency Coupling

In Data-1, delta power, calculated using a Fast Fourier Transform applied every second and then averaged for the entire duration of the selected segment, was increased in stage N3 relative to stage N2 (47.3 ± 12.6 vs. 22.8 ± 3.9 μV2/Hz; *p*: 0.001). Periods of continuous vs. discontinuous slow oscillation had increased absolute delta power (51.6 ± 27.8 vs. 31.7 ± 6.3 μV2/Hz, *p*: 0.02) in Data-1 and Data-2 (78 ± 15.2 vs. 42.8 ± 13.9 μV2/Hz; *p*: 0.001).

### Beat-to-Beat Blood Pressure and Sleep in Data 2 (Table 3)

**FIGURE 4:**
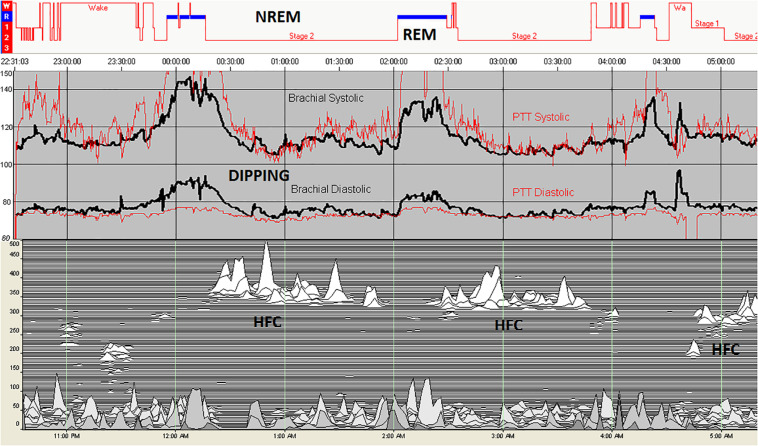
Blood pressure in relation to sleep stage and state. Intra-arterial (black) and PTT-derived (red) blood pressure during polysomnography shows a rise during REM sleep and reductions during periods of high frequency coupling during stage N2. In this individual, there is no N3 sleep, demonstrating the independence of high frequency coupling from conventional slow wave sleep. REM sleep results in an increase in blood pressure. The drop of blood pressure in the initial wake periods reflected the effect of quiet/relaxed state on cardiovascular hemodynamics. HFC, high frequency coupling periods; NREM, non-rapid eye movement; and REM, rapid eye movement.

For a total of 11 subjects, a total of 5832 epochs (2916 min) of artifact-free conventional sleep were analyzed (N1:386, N2:3177, N3:1272, REM: 997). Based on ECG-spectrogram analysis, 1875 interpolated epochs were HFC, vs. 3901 were non-HFC. The total spectrographic analysis was 5776 epochs, which is slightly less than the number of conventional stage epochs as the spectrographic windows are 2.1 min, needing a rounding off described above. A within-subjects repeated measures Analysis of Variance of the Finometer/intra-arterial was performed using epoch (of state or stage) as the repeated factor. N3 had the lowest blood pressure of the conventional stages [*F*(_3_, _5818_): 90.05, *p* < 0.001]. High frequency coupling [*F*(_1_, _5807_): 218.7, *p* < 0.001], regardless of conventional stages was associated with reduced mean blood pressure (Figure 4).

**TABLE 3:** (Data-2): Beat-to-beat blood pressure in relation to sleep stage and state (11 subjects).

**Stage, state**	**PTT systolic**	**Finometer systolic**	**PTT diastolic**	**Finometer diastolic**	**Heart rate**
N1	119.2 ± 16.8	117 ± 18.8	68.6 ± 8.9	60.7 ± 13.2	67.2 ± 17.8
N2	112.1 ± 16.4	109.8 ± 18.6	66.8 ± 10.1	57.5 ± 13.9	59.2 ± 15.8
N3	103.9 ± 10.9	103.5 ± 15.4	64.3 ± 8.8	54 ± 13.8	55.2 ± 10.1
REM	110.2 ± 19.4	109.1 ± 16.9	66.7 ± 11.6	58.3 ± 15.1	60.2 ± 13
HFC	108.7 ± 14.7	108.8 ± 18.5	65.1 ± 10.5	57 ± 14.8	64.4 ± 15
Non-HFC	114.7 ± 18.3	116 ± 16.9	68.9 ± 8.5	62.4 ± 13.8	55.9 ± 14.1

*N1, N2, N3, non-rapid eye movement stages; REM: rapid eye movement; HFC: high frequency coupling; CPC: cardiopulmonary coupling; and PTT: pulse transit time.*

### Unstable REM Sleep – Data 3, Figure 5

For a total of 50 subjects with high loop gain apnea, the subject population characteristics were 37/50 males, BMI 32.3 ± 6.7 Kg/M2, age: 54.3 ± 8 years. There was a total of 127 REM periods of at least 15 min duration (mean: 22.2 ± 7.1 min). In 78/127 (61.4%) periods, a segment of unstable REM across EEG and respiratory domains, were noted. These occurred exclusively at the transition into REM sleep, never at the end of a REM period. In 12 REM periods, periodic breathing occurred at the edge of EEG-based REM sleep before converting into typical REM apnea.

**FIGURE 5:**
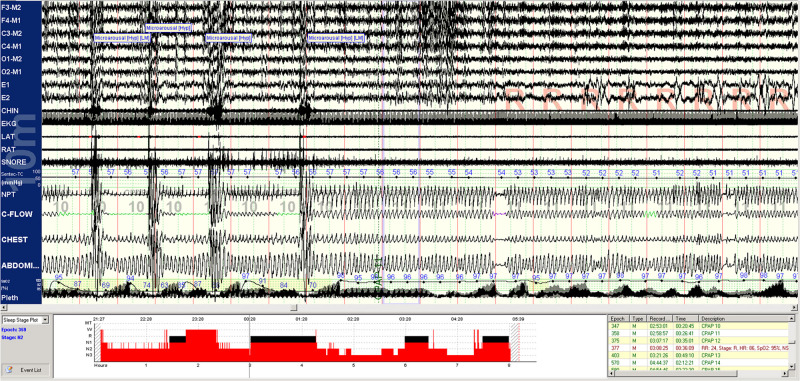
REM breathing in NREM sleep. Periodic breathing with mild obstructive features is unresponsive to CPAP in NREM sleep. Approximately 2.5 min before the onset of classic REM sleep, respiration abruptly stabilizes and remains stable for the rest of REM sleep. Stabilization begins before the increase of CPAP from 10 to 11 cms H_2_O. EEG montage is standard. EKG, electrocardiogram; SNORE, snoring microphone; LAT, left anterior tibial electromyogram; RAT, right anterior tibial electromyogram; and NPT, nasal pressure (mask pressure). C-FLOW: flow signal from the CPAP machine; CHEST: chest effort; ABDOMI: abdominal effort Pleth: singer pulse plethysmogram.

**FIGURE 6:**
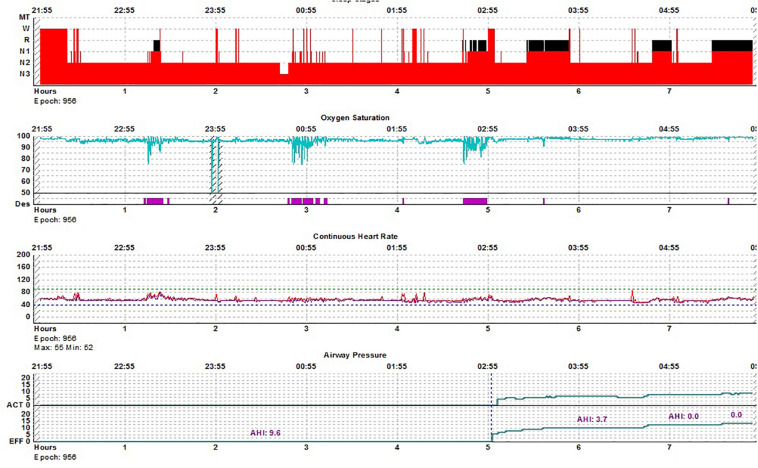
REM breathing in NREM sleep-hypnogram. Split-night polysomnogram showing REM-dominant sleep apnea. The second REM period is not seen on the surface conventional scoring yet demonstrates the classic desaturation pattern of REM sleep.

### Gender Differences

Across all analyses, no statistically significant gender differences were noted.

## Discussion

The key results are as follows: (1) A bimodal pattern of NREM and REM sleep was evident using a multi-physiology approach. (2) The NREM sleep slow oscillation shows two modes, one mode dominated by nearly continuous slow waves/down states (conventional N3 and most of healthy N2) and another with intermittent slow waves/down states (K-complexes by conventional characterization; rarely N3, usually N2). (3) Blood pressure during sleep was lowest during stage N3, slow wave sleep. (3) Blood pressure was reduced during periods of HFC during subsets of N2 that exhibited HFC characteristics, similar to that seen during N3. (4) Utilizing respiratory patterns distinct to obstructive apnea and periodic breathing, unstable REM (or REM_US_) was discernable.

Non-rapid eye movement sleep bimodality is readily visible in polysomnographic data. Delta power covaries with the high frequency component of HRV (Yang et al., 2002; Rothenberger et al., 2014). However, the evolution of HRV power across the sleep period and in relation to stages does not show sharp boundaries. Windowing HRV through respiration and respiratory control systems, which is what the CPC technique achieves, enables clear boundary distinctions of respiratory-autonomic-sleep integration. Viewing sleep through this lens, the results presented support the concept of bimodality rather than the multi-step depth approach to characterizing NREM sleep (Thomas and Mietus, 2011). The continuous mode of the slow oscillation was enriched during periods of HFC, a state we have previously shown to be correlated with delta power and requires stable breathing to be detected computationally (Thomas et al., 2014). If blood pressure dipping occurs only during these same periods, then periods of HFC simultaneously also demonstrate stable breathing, high delta power, and vagal HRV dominance. These are the properties we propose make up NREM_S_. As periods of HFC and LFC alternate and abruptly switch across the whole sleep period during NREM sleep, these results show a dimension and characterization of NREM sleep not previously recognized, but reminiscent of what has previously been called NREM “packets” (Watson et al., 2016). While the EEG is information rich, these “packets” of NREM sleep with desirable qualities may capture a complementary read-out of sleep physiology and quality.

Even in conditions associated with fragmented sleep, such as sleep apnea, delta power and vagal HRV dominance tends to ebb and flow in a correlated manner (Jurysta et al., 2006). How do cortical phenomena impact subcortical and brainstem function such that integrated multi-component states exist? Two possibilities are proposed. In one, the <1 Hz slow oscillation, which can be recorded in subcortical and brain stem neural sub-systems (Eschenko et al., 2012), provides a global synchronizing influence. The impact of cortical down states is possibly far more widespread across the neuraxis, beyond the characterization in the thalamus and striatum. Such influences would enable stable breathing, blood pressure dipping, vagal autonomic dominance, and high delta power to occur simultaneously. In another scenario, the thalamus may provide a multi-loop synchronizing function (Crunelli et al., 2014).

Our results on blood pressure across states of sleep reflects vertical integration of multiple physiologies from the cortex to the vasculature. A reduction in blood pressure during sleep (“dipping”) is considered a blood pressure-related biomarker of healthy sleep (Routledge et al., 2007; Salles et al., 2016). There is a progressive reduction of blood pressure from wake through slow wave sleep, with an increase in REM sleep or transiently in association with arousals. Ambulatory blood pressure measurement using intermittent cuff inflation has shown the amount of slow wave sleep to correlate with dipping (Sayk et al., 2010; Javaheri and Redline, 2012). We show that blood pressure reduction during sleep occurs only during periods of high frequency coupling, our proposed biomarker of NREM_S_. This correlation was observed independent of N3 sleep. The results strengthen the concept that the NREM slow oscillation has profound effects across the neuraxis, binding cortical, subcortical and brainstem mechanisms to form a state of sleep that can perform its functions, not well-captured by standard EEG-based staging.

The association of blood pressure dipping exclusively with NREM_S_ is important as NREM_S_ is in turn associated with a continuous mode of expression of the NREM slow oscillation. Non-dipping of blood pressure could in theory be caused by pathology at multiple levels of the system, from the cortex all the way down to the endothelium and arterioles. Conditions and stimuli that increase fragmentation of the slow oscillation, such as arousals (of which there are numerous causes) and intrinsic diseases such as neurodegeneration (Alzheimer’s disease, Parkinson’s disease, stroke) may alter hemodynamic profiles during sleep and impose a vascular burden on cortical health. Treatment of sleep fragmenting conditions can improve sleep hemodynamics. It is also possible that drugs which enhance high frequency coupling, such as benzodiazepines (Thomas et al., 2017) may benefit sleep hemodynamics, though other undesirable effects alter the risk benefit balance and may limit general use.

Rapid eye movement sleep bimodality is less intuitive but in retrospect should have been identified earlier. Transitions to and from REM sleep have elaborate rules and are often the areas where the lowest inter-scorer reliability occurs, especially N2 to REM (Rosenberg and Van Hout, 2013). Dissociated states where the distinction of REM and NREM is difficult or impossible are well described (Mahowald and Schenck, 1991; Vetrugno et al., 2009; Vetrugno and Montagna, 2011; Abgrall et al., 2015; Antelmi et al., 2016). Blurring of sleep states have been described in narcolepsy, where state instability is an important pathology (Diniz Behn et al., 2010; Olsen et al., 2017). Our data adds respiratory patterns to this analysis, and show that such transitional states, where there are features of coexisting REM and NREM sleep, are relatively common. Recognizing this transitional state as unstable REM or REM_US_ can provide insights into diseases where REM pathology may be of interest, such as post-traumatic stress disorder, REM behavior disorder, neurodegeneration in general, narcolepsy and sleep apnea.

Our study has some limitations. The EEG montages used, typical of clinical and research PSG, did not allow estimation of local vs. global slow wave activity (Nobili et al., 2012; Timofeev, 2013). Recording beat-to-beat blood pressure with high density EEG will be required to determine the relationship of cortical slow wave activity, CPC, and blood pressure at a finer resolution that we have been able to show. The standard filter settings on PSG also impose a cut-off at 0.3 Hz, filtering out some components of the <1 Hz slow oscillation (clinical PSG does not use a full-band EEG approach). We did not study children, who have an abundance of slow wave activity, or the elderly, who have reduced slow wave activity. However, HFC dominates healthy sleep in children (Lee et al., 2012; Cysarz et al., 2018) and adults including the elderly (Thomas et al., 2009).

In summary, we show that both NREM and REM sleep have cross-modal bimodal characteristics, with concordant occurrence of stable breathing, high delta power, a consolidated <1 Hz slow oscillation dominated by frequent cortical down states, and blood pressure dipping as a feature of an integrated “stable” NREM sleep. Readily recognizable admixtures of REM and NREM physiology are common in sleep apnea patients and provide support to the concept of REM bimodality. Such bimodal states are entirely consistent with the features of sleep described by network physiologists. Our results also point to the importance of using a multi-physiology approach to identify sleep states, than relying exclusively on EEG-based staging. Our results and those of other complementary sleep typing systems may also motivate a new look at neurocircuitry models of sleep regulation.

## Data Availability Statement

The raw data supporting the conclusions of this article will be made available by the authors, without undue reservation.

## Ethics Statement

The studies involving human participants were reviewed and approved by Institutional Review Board/Committee on Clinical Investigations, Beth Israel Deaconess Medical Center, Boston. The patients/participants provided their written informed consent to participate in this study.

## Author Contributions

CW: Data collection, curation, tabulation, analysis, and manuscript review. MB: Theory conceptualization, manuscript writing, and editing and review. C-HY: Theory conceptualization, manuscript writing and review. CS: Theory conceptualization, manuscript writing and review. RT: Software development (cardiopulmonary coupling), theory conceptualization, data analysis, and manuscript writing and editing. All authors contributed to the article and approved the submitted version.

## Conflict of Interest

RT: (1) Patent for a device to regulate CO_2_ in the positive airway pressure circuit, for treatment of central/complex apnea. (2) Patent and license for an ECG-based method to phenotype sleep quality and sleep apnea (to MyCardio, LLC, through Beth Israel Deaconess Medical Center). (3) Patent, past consultant – DeVilbiss-Drive, CPAP auto-titrating algorithm. (4) GLG Councils and Guidepoint Global– general sleep medicine consulting. MB has received funding in the past from the Department of Neurology, Massachusetts General Hospital, the Center for Integration of Medicine and Innovative. Technology, the Milton Family Foundation, the MGH-MIT Grand Challenge, and the American Sleep Medicine Foundation. MB has had research contracts with MC10 and Insomnisolv, consulting agreements with McKesson, International Flavors and Fragrances, and Apple Inc., and has served as a medical monitor for Pfizer. MB has received payment for educational material from Oakstone Publishing and has provided expert testimony in sleep medicine. None of these entities were involved in the present work. The remaining authors declare that the research was conducted in the absence of any commercial or financial relationships that could be construed as a potential conflict of interest.

## Footnotes

1 http://www.aasmnet.org/scoringmanual/default.aspx
